# ISSD: Improved SSD for Insulator and Spacer Online Detection Based on UAV System

**DOI:** 10.3390/s20236961

**Published:** 2020-12-05

**Authors:** Xuan Liu, Yong Li, Feng Shuang, Fang Gao, Xiang Zhou, Xingzhi Chen

**Affiliations:** College of Electrical Engineering, Guangxi University, Nanning 530000, China; 1812392024@st.gxu.edu.cn (X.L.); fshuang@gxu.edu.cn (F.S.); fgao@gxu.edu.cn (F.G.); 1812401013@st.gxu.edu.cn (X.Z.); 1812392005@st.gxu.edu.cn (X.C.)

**Keywords:** SSD, insulator, spacer, object detection, UAV, power inspection

## Abstract

In power inspection tasks, the insulator and spacer are important inspection objects. UAV (unmanned aerial vehicle) power inspection is becoming more and more popular. However, due to the limited computing resources carried by a UAV, a lighter model with small model size, high detection accuracy, and fast detection speed is needed to achieve online detection. In order to realize the online detection of power inspection objects, we propose an improved SSD (single shot multibox detector) insulator and spacer detection algorithm using the power inspection images collected by a UAV. In the proposed algorithm, the lightweight network MnasNet is used as the feature extraction network to generate feature maps. Then, two multiscale feature fusion methods are used to fuse multiple feature maps. Lastly, a power inspection object dataset containing insulators and spacers based on aerial images is built, and the performance of the proposed algorithm is tested on real aerial images and videos. Experimental results show that the proposed algorithm can efficiently detect insulators and spacers. Compared with existing algorithms, the proposed algorithm has the advantages of small model size and fast detection speed. The detection accuracy can achieve 93.8%. The detection time of a single image on TX2 (NVIDIA Jetson TX2) is 154 ms and the capture rate on TX2 is 8.27 fps, which allows realizing online detection.

## 1. Introduction

**Figure 1:**
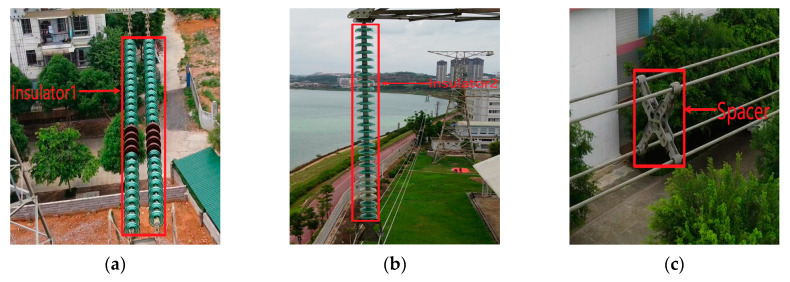
Electrical components: (**a**) Insulator1; (**b**) Insulator2; (**c**) Spacer.

The insulator and spacer are important components of power transmission lines. The insulator can achieve electrical insulation and mechanical fixation. The spacer can fix the spacing between split wires, prevent whipping between wires, and suppress breeze vibration. The health status of insulators and spacers has an important impact on the stable and safe operation of high-voltage transmission lines [1]. Thus, it is necessary to periodically inspect the transmission line components, e.g., insulators and spacers. Figure 1 shows some electrical components on the transmission line. Figure 1a is the first type of insulator, named Insulator1. Figure 1b is the second type of insulator, named Insulator2. Figure 1c is the four-split spacer, named Spacer. At present, the common transmission line inspection methods mainly include manual inspection, robot-based inspection, and unmanned aerial vehicle (UAV)-based inspection [2,3]. Manual inspection of electric power exposes the inspection staff to certain dangers and the operation efficiency is not high. Robot-based inspection needs a specific mechanical structure to move on the wires and cross obstacles such as antivibration hammers [4,5,6]. A specific device is required for the robot to go up and down the tower, and the process is complicated, while the robot′s inspection efficiency is low. UAVs are flexible and have high inspection efficiency [7,8], and they are widely used in daily inspections. 

At present, UAV power inspection usually uses the camera carried by the UAV to collect images of the transmission lines and their key components. For the inspection, the power components need to be detected in real time. Then, the state of the power component, i.e., normal or damaged, is judged. In the power inspection, accurate and effective inspection object detection is one of the key technologies. In recent years, with the rapid development of deep learning, researchers in the power field have also begun applying deep learning techniques into the detection of power inspection objects. Heng et al. [9] proposed a method for antivibration hammer detection. This method uses faster R-CNN (region-based convolutional neural network) [10] for antivibration hammer recognition and location extraction in aerial images. However, the accuracy of this method is relatively low and the detection speed is slow. To improve the detection accuracy and speed, Chen et al. [11] proposed a novel method to identify insulators and antivibration hammers. In this method, an SR-CNN (super-resolution convolutional neural network) [12] is used to reconstruct a blurred image with high resolution, and then the YOLOv3 (you only look once) [13] network is used to recognize the reconstructed image. For overhead distribution line insulator detection, Prates et al. [14] proposed a deep learning-based method, which uses the VGG [15] network to classify the insulator images. However, this method does not detect the position of insulators. After that, Jiang et al. [16] used MobileNet [17] as the backbone of SSD (single shot multibox detector) [18] to detect missing caps, which represent the most frequent fault of insulators. Here, low-level, middle-level, and high-level feature maps are used for insulator detection, which can improve the detection accuracy. Additionally, Hu et al. [19] used the improved faster R-CNN to detect railway catenary insulators. This method adds an angle of object during training, which makes the detection box closer to the object. However, this model has a larger weight, which can take up more memory space. Han et al. [20] proposed a cascaded model to detect multiple insulator faults in the aerial images. This method used the YOLOv3 tiny network to detect insulators. Most of these methods were tested on high-performance computers without testing on embedded devices. Thus, it is difficult to judge whether the methods meet the real-time requirements of UAVs for data processing.

The existing object detection algorithms for power inspection can be divided into two-stage detection frameworks (e.g., Faster R-CNN [10], R-FCN (region-based fully convolutional networks) [21]) and one-stage detection frameworks (e.g., SSD [18]). The detection speed of the two-stage detection framework is usually slower than that of the one-stage detection framework, and the model weight size is usually greater than the one-stage detection framework. As we know, the embedded device carried by the UAV has limited computing resources and small memory space. Thus, the two-stage detection framework is usually not suitable for deployment on UAVs equipped with embedded devices to achieve online inspection. In order to achieve effective online insulator and spacer detection, we first construct a new insulator and spacer detection dataset, named ISD-dataset. Then, we propose an improved SSD algorithm, named ISSD. The improved algorithm uses MnasNet [22] as the main feature extraction network to generate feature maps. Next, the multiscale feature maps are fused and then the detection results are generated through NMS (non-maximum suppression). Finally, the detection speed is tested on a TX2 (NVIDIA Jetson TX2) and server. The test results show that the proposed algorithm can meet the real-time requirements on a server and can be deployed on TX2 to realize online detection [23,24].

In summary, the main contributions of this paper are as follows:

(1) A new dataset named ISD-dataset is constructed, which can be used for insulator and spacer detection through aerial images. The ISD-dataset contains three types of objects, i.e., Insulator1, Insulator2, and spacer, which allows effectively testing the performance of the object detection algorithm for UAV power inspection.
(2) An improved SSD algorithm for UAV online detection of insulator and spacer is proposed. The improved algorithm uses a lightweight network, i.e., MnasNet, as the feature extraction network, which improves the feature extraction capability and reduces the amount of calculation and model size. This network can generate feature maps with rich features more quickly. Then, two multiscale feature fusion methods, i.e., Channel plus and Channel concatenate, are used to fuse low-level features and high-level features to improve the detection accuracy. The proposed algorithm is superior to previous algorithms in terms of accuracy and speed.
(3) We test the performance of the proposed algorithm, faster R-CNN, R-FCN, and SSD on the ISD-dataset. The comparison of experimental results proves that the proposed algorithm has the advantages of being a small model with high accuracy and fast detection speed. The test results on TX2 show that the proposed algorithm is more suitable for deployment on mobile devices (e.g., TX2). The online detection results of video can be found at https://github.com/PromiseXuan/Insulator-detection.

## 2. Related Works

### 2.1. Faster R-CNN

Faster R-CNN [10] is a two-stage framework. This network constructs the region proposals first, and then classifies the selected regions. Faster R-CNN uses a set of basic convolutional networks to extract the feature map of the image. Two branches are formed on the feature map. One branch uses the feature map to generate region proposals through RPNs (region proposal networks). Another branch sends the feature map and region proposal to the ROI (region of interest) pooling layer to generate the proposal feature map. Finally, the object category and the location of the detection box on the proposal feature maps are calculated. Faster R-CNN uses RPNs to generate region proposals, which improves detection speed and accuracy. However, after ROI pooling, each region connects with multiple fully connected layers, which requires a large amount of parameter calculation. This leads to a relatively slow detection speed. Faster R-CNN needs to train RPNs and classification networks. The faster R-CNN model size is large, which takes up more computing resources. Furthermore, the performance of faster R-CNN for small object detection needs to be improved.

### 2.2. R-FCN

R-FCN [21] is also a two-stage framework. The R-FCN network consists of shared fully convolutional structures and does not contain a fully connected layer. In R-FCN, position-sensitive score maps are proposed to solve the contradiction problem of translation invariance and translation variance in object detection. There are two branches on the feature map obtained by the last convolutional layer of the feature extraction network. A branch is sent to the RPN to generate a region proposal. Another branch is sent to the convolutional layer to generate position-sensitive score maps. Finally, the corresponding category and location information can be obtained through ROI pooling. Compared with faster R-CNN, R-FCN removes the fully connected layer and reduces the amount of calculation, which increases the detection speed. However, because it is still a two-stage framework network, the detection time is also longer.

### 2.3. SSD

SSD [18] is a one-stage framework for object detection. The basic idea is that an image is input into the network, and then a reasonable output vector is set. Next, the regression approach is used to output the object′s bounding box and category confidence. SSD produces a fixed-size collection of bounding boxes and scores for those boxes, which exist as object class instances. Finally, NMS is used to produce the final result. SSD uses multiscale feature maps for object detection, which improves the detection accuracy of small objects.

**Figure 2:**
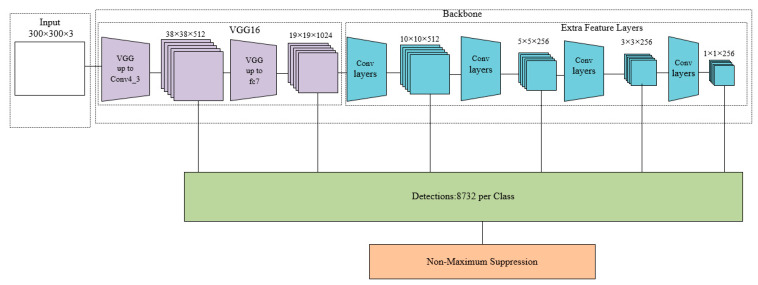
Single shot multibox detector (SSD) network.

As shown in Figure 2, the SSD network consists of four parts. The first part is the image input module. The input image size can be 300 × 300 or 512 × 512. The second part is the feature extraction network. SSD uses VGG16 [15], which is truncated before the classification layer, as the basic model. After that, new convolutional layers are added to obtain more feature maps for object detection. The third part is the detection layer. It uses a 3 × 3 convolution for each feature map to generate the confidence of each category and the offset of the predicted bounding box relative to the default box. The detection result contains two parts, i.e., category confidence and bounding box. The fourth part is NMS. The redundant detection boxes are removed and the detection boxes having the highest intersection over union with ground truth are retained to obtain the final detection result. However, the larger network model is not suitable for deployment on small mobile devices.

## 3. Proposed Method

**Figure 3:**
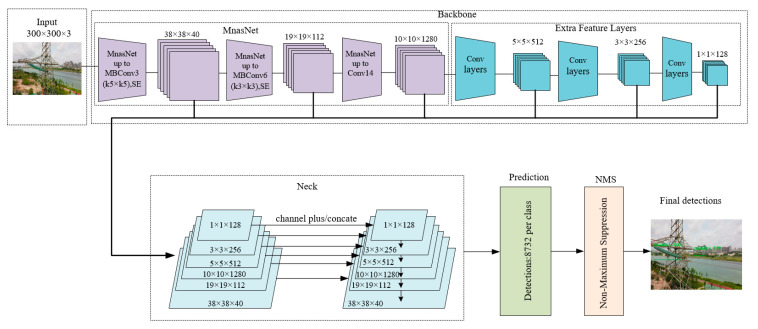
Improved SSD network (ISSD).

The proposed network mainly includes five parts, i.e., input, backbone, neck, prediction, and NMS. Figure 3 shows the proposed network. Input denotes the input image, which normalizes the image size to 300 × 300 × 3. Backbone is a feature extraction network, which is used to generate feature maps to obtain image features. Neck is a multiscale feature fusion module, which combines low-level feature maps and high-level feature maps to generate new feature maps for object prediction. Prediction is the object prediction module, which performs a convolution operation on the feature map obtained by the neck to obtain the predicted bounding box and category confidence. NMS is used to remove redundant detection boxes.

### 3.1. Backbone

Backbone is a feature extraction network for feature map generation. As shown in Figure 3, the backbone consists of two parts, i.e., MnasNet and extra feature layers.

To run a model on mobile devices with limited computing resources and storage resources, we need to pay attention to three indicators of the model, i.e., model size, inference speed, and detection accuracy. MnasNet is a convolutional neural network model, which is suitable for mobile devices obtained using the neural architecture search approach. It takes the inference time of the model on the mobile device as the main optimization goal to optimize the model, allowing a good balance between accuracy and speed. The main network structure of MnasNet is shown in the upper left part of Figure 3. The objective function of MnasNet is shown in Equations (1) and (2), where *n* is the target model, *Acc(n)* represents the accuracy of the target model, *Lat(n)* represents the actual inference time of the target model on the mobile device, *T* is the target inference time, and *y* is a weight factor. When the actual inference time *Lat(n)* is less than the target inference time *T*, *y = a*; otherwise, *y = b*. When *a = b* = −0.07, MnasNet can achieve a good balance between accuracy and speed.

(1) $$\underset{n}{\max}\;Acc(n)\times {\left[\frac{Lat\left(n\right)}{T}\right]}^{y}.$$

(2) $$y=\{\begin{array}{l} a,\;if\;Lat(n)<T\\ b,\;otherwise \end{array}.$$

In terms of model size, the parameter size of VGG16 is 138 M, while the parameter size of MnasNet is 3.9 M. On the ImageNet [25] benchmark, the accuracy of MnasNet is higher than that of VGG16. MnasNet has few parameters, fast speed, low delay, and high recognition rate, making it more suitable for use on embedded devices. In order to provide MnasNet with better feature extraction capability, transfer learning is employed. The pretrained model parameters are used as the initial parameters of the feature extraction network in the proposed method.

In order to strengthen the feature extraction capability of the network, extra feature layers are added after MnasNet. As shown in the upper right of Figure 3, the extra feature layers constitute three convolutional layers. The convolution calculation is shown in Equations (3) and (4), and the output feature map size is calculated according to Equation (5), where *G_i_* is the feature map of the *i*-th layer, *W_i_* and *B_i_* are the weight vector and offset vector of the *i*-th layer convolution kernel, respectively, *F()* is the activation function, *H_i_* and *O_i_* are the height and width of the feature map, and *k*, *q*, and *s* are the size, padding, and step length of the convolution kernel, respectively. After the feature map undergoes a convolution with a step length of 2, the size of the feature map is reduced to half of the original size, and the receptive field can be expanded, which can provide low/intermediate information supplementation for the standard pretrained network. This contributes to the detection of small and medium objects.

(3) $$G_{i}=F\left(W_{i}⊗G_{i-1}+B_{i}\right).$$

(4) $$F(x)=\{\begin{array}{ll} x, & x>0\\ 0, & \mathrm{otherwise} \end{array}.$$

(5) $$H_{i}=\left(\frac{H_{i-1}-k_{i}+2q_{i}}{s_{i}}+1\right)\;O_{i}=\left(\frac{O_{i-1}-k_{i}+2q_{i}}{s_{i}}+1\right).$$

### 3.2. Neck

**Figure 4:**
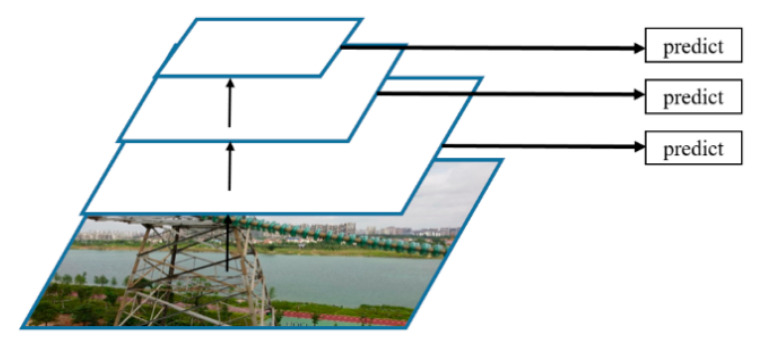
Multiscale prediction module in SSD.

In order to make the algorithm more adaptable to object detection of different sizes, low-level features and high-level features are usually simultaneously used. Since the characteristics of different objects have certain differences, shallow-level features can distinguish simple objects, and depth-level features are conducive to distinguishing complex objects. Figure 4 shows the modules of multiscale prediction in the SSD network. This structure can make predictions on feature maps of different sizes and improve the performance of the detection algorithm. However, the features extracted by this structure for object prediction are not robust enough. Most of the features are obtained from a shallower layer, and the used features are from the same layer.

**Figure 5:**
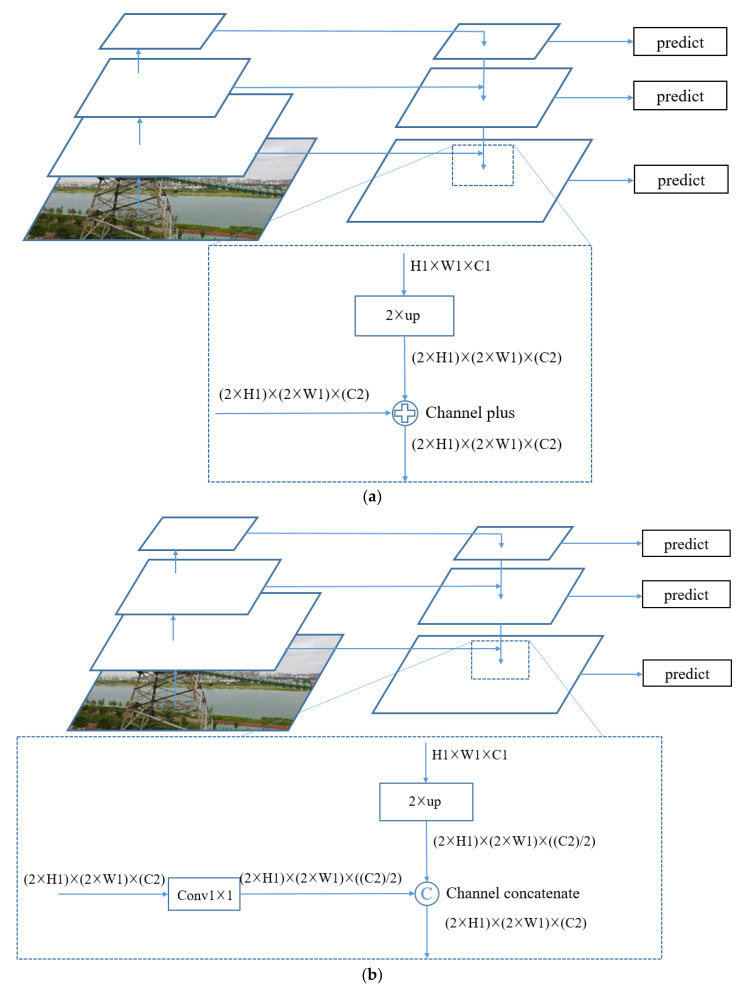
Neck: (**a**) Channel plus; (**b**) Channel concatenate.

In order to make the features used in object prediction more abundant and robust, we draw on the idea of FPN (Feature Pyramid Networks) [26] to propose two feature fusion methods, as shown in Figure 5. Figure 5a is the first method denoted as Channel plus. In this method, the high-level feature map is up-sampled so that the width *O_1_*, the height *H_1_*, and the number of channels *C_1_* of the high-level feature map are the same size as those of the low-level feature map. Then, the values of the corresponding positions of the two feature maps are directly added to obtain the fused feature map. The calculation method is shown in Equation (6), where *z_l3_* is the feature vector of the feature map *l_3_* after fusion, *f_l1_* is the feature vector of the high-level feature map *l*_1_, and *f_l_*_2_ is the feature vector of the low-level feature map *l_2_*. Here, the channel numbers of *l*_1_ and *l*_2_ are equal.

(6) $$z_{l3}=f_{l1}+f_{l2}.$$

Figure 5b shows the flowchart of the second method, which is named Channel concatenate. First, the high-level feature map is up-sampled to make the width and height consistent with the low-level feature map, and the number of channels is half that for the low-level feature map. Then the high-level feature map *C_l*1*_* can be generated. At the same time, a convolution operation is performed on the low-level feature map with a convolution kernel of 1 × 1, and the number of channels of the low-level feature map is changed to half of the original to obtain the low-level feature map *C_l_*_2_. The two feature maps are concatenated on the basis of the channels to obtain the fused feature map *C_l*3*_*. The calculation method is shown in Equation (7), where *concatenate(·,·)* is the channel concatenate function.

(7) $$C_{l3}=concatenate(C_{l1},C_{l2}).$$

Through these two methods, high-level features and low-level features are fused to construct a deeper feature pyramid, which can integrate multilevel feature information and perform output prediction on different feature maps.

### 3.3. Loss Function and Optimization

The loss function of the network consists of two parts, i.e., the position error and the category confidence error. As shown in Equation (8), the total objective loss function is the weighted sum of the position error and the confidence error.

(8) $$L(t,p,u,v)=\frac{1}{S}\left(L_{cls}(t,p)+\lambda L_{box}(t,u,v)\right),$$

where *S* is the number of matched default boxes, *t* = {0,1} (when the *i*-th default box matches with the *j*-th ground truth, *t* is 1; otherwise, *t* is 0), *p* is the category confidence, *u* is the predicted value of the bounding box offsets, *v* is the ground truth, and *λ* is a hyperparameter, which is the weight coefficient of position error loss and category error loss. Here, we set *λ =* 1.

The position error loss function *L_box_* is shown in Equations (9)–(12), where *(o, r, w, h)* represent the center point coordinate of the box and the length and width of the box. When the *i*-th default box and the *j*-th ground truth box can match, *t =* 1. We only calculate the position error loss of boxes which present object instances. Positive denotes that the detection box contains an object.

(9) $$L_{box}(t,u,v)=\sum_{i\in Positive}^{S}\sum_{m\in \{o,r,w,h\}}^{}t_{ij}F\left(u_{i}^{m}-{\tilde{v}}_{j}^{m}\right).$$

(10) $$F\left(e\right)=\{\begin{array}{ll} 0.5e^{2} & if\;\left|e\right|\;<1\\ \left|e\right|-0.5 & otherwise \end{array}.$$

(11) $${\tilde{v}}_{j}^{o}=\left(v_{j}^{o}-d_{i}^{o}\right)/d_{i}^{w}\;{\tilde{v}}_{j}^{r}=\left(v_{j}^{r}-d_{i}^{r}\right)/d_{i}^{r}.$$

(12) $${\tilde{v}}_{j}^{w}=\log\left(v_{j}^{w}/d_{i}^{w}\right)\;{\tilde{v}}_{j}^{h}=\log\left(v_{j}^{h}/d_{i}^{h}\right).$$

As shown in Equations (13) and (14), *L_cls_(t,p)* is the category confidence loss function. When calculating category confidence errors, both positive and negative samples must be calculated. *k* represents the *k*-th object. Negative denotes that there is no object in the detection box

(13) $$L_{cls}(t,p)=-\left(\sum_{i\in Positive}^{S}t_{ij}^{k}\log\left({\tilde{p}}_{i}^{k}\right)+\sum_{i\in \mathrm{Negative}}\log\left({\tilde{p}}_{i}^{0}\right)\right).$$

(14) $${\tilde{p}}_{i}^{k}=\frac{\exp\left(p_{i}^{k}\right)}{\sum_{k}\exp\left(p_{i}^{k}\right)}.$$

The Adam [27] optimization algorithm is used to optimize the network, which can achieve the improvement of optimization quality and speed by obtaining an adaptive learning rate of each parameter during the model training optimization process.

## 4. Experiments

### 4.1. Offline Detection versus Online Detection

**Figure 6:**
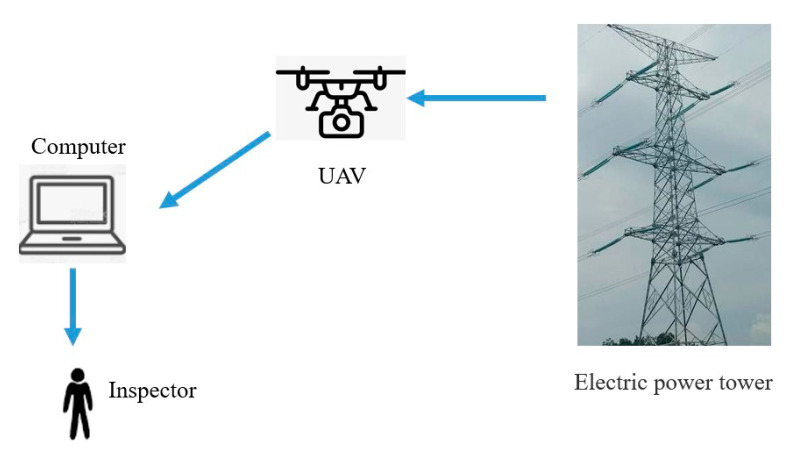
Offline detection.

Most UAV-based transmission line inspection systems use offline inspection schemes. As shown in Figure 6, the UAV captures video with a camera, and then transmits it to a local computer or data processing center. Inspectors inspect the video and judge the status of the power components in the video.

**Figure 7:**
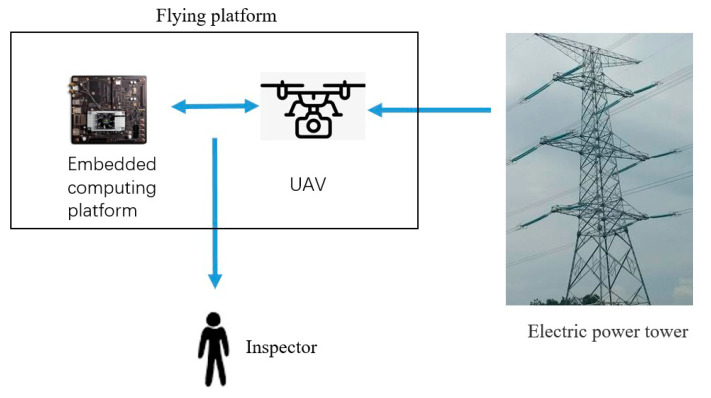
Online detection.

We propose an online detection method. As shown in Figure 7, the UAV captures the image and transmits it to the onboard embedded processor TX2 for inspection. When an object is detected, the UAV adjusts the flight attitude according to the relative position of the object in the image so that object is in the center of the image. This clarifies the image of the detection object.

In offline detection, the UAV is only used to capture video. Offline detection results in a need to store the video during the entire inspection process. Online detection allows only storing images containing the detection object. Online inspection can improve the inspection efficiency and reduce required storage space.

### 4.2. UAV Platform

**Figure 8:**
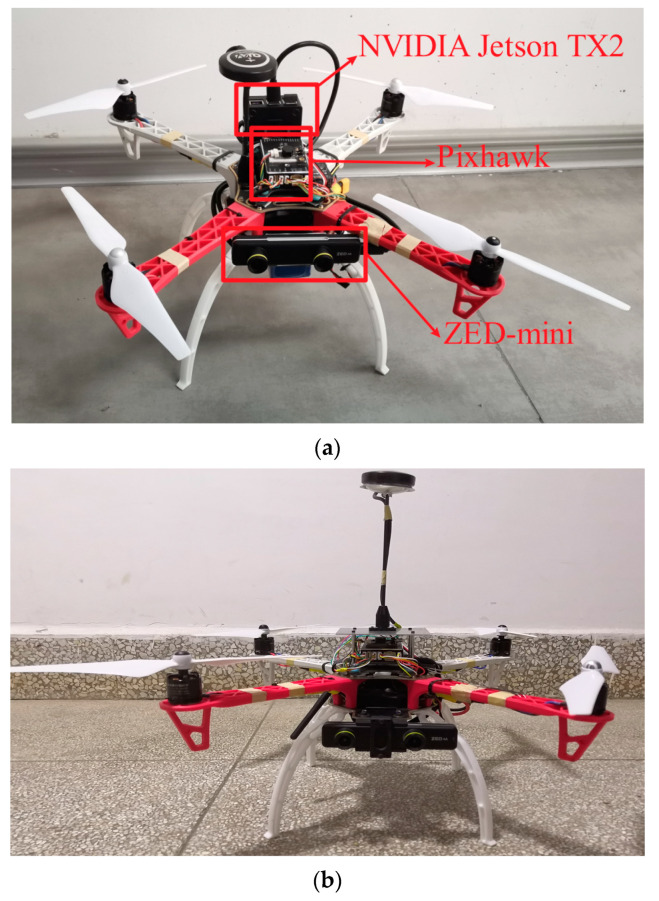
Unmanned aerial vehicle (UAV) platform. (**a**) Top view, (**b**) Front view.

In order to realize online detection of high-voltage transmission lines, we built a custom UAV platform (shown in Figure 8). The UAV is based on an F450 quadrotor airframe, equipped with a Pixhawk running ArduPilot firmware as the autopilot and an XBee radio module for GCS (ground control station) telemetry. The UAV is powered by one 5000 mAh, 22.2 V, 25 C, 6 S1P lithium polymer (Li–po) battery pack, which allows it to fly up to 35 min and reach an average speed of 60 km/h.

The primary payload of the UAV is an embedded AI (artificial intelligence) system TX2, which is connected to a ZED Mini camera. The ZED Mini is a stereo camera. It is able to generate red/green/blue (RGB) images and corresponding three-dimensional (3D) point clouds. Since the coordinate origin of point clouds is set to the optical center of the left camera, we only used the left-view RGB image for target detection. In the future, we will combine 3D point clouds for UAV navigation and obstacle avoidance. The ZED Mini camera is rigidly mounted on the front part of UAV to capture forward-looking images.

### 4.3. Dataset

We kept a distance of 1.5 m to 2.0 m between the UAV and the power tower for shooting. In the insulator string, the diameter of each insulator was 280 mm. The length of the four-split spacer was 500 mm. The resolution of the captured image was 1920 × 1080, and a total of 1500 aerial images containing power components were selected.

To make the ground truth of the object detection dataset, we used Labelimg (https://github.com/tzutalin/labelImg) to label the insulators and spacers in the aerial images of various types of power poles and towers. Then, we built a Pascal VOC (Visual Object Classes) [28] format dataset named the ISD-dataset. In the ISD-dataset, insulator and spacer objects are included. According to the installation method of the insulator, the insulators are classified as Insulator1 or Insulator2. The number of various objects contained in the ISD-dataset is shown in Table 1. We randomly selected 70% of the images in the dataset as the training set and 30% of the images as the test set. In the training stage, random cropping, random left and right mirroring, etc. were used to enhance the data.

### 4.4. Experimental Setup

The experimental environment and configuration were as follows:

Server configuration: central processing unit (CPU) model, Intel(R) Xeon(R) Gold 6136; graphics processing unit (GPU) model, 8× TITAN V. CUDA version 10.2; operating system, Ubuntu 16.04; Pytorch [29] version 1.3.0.

Embedded device: GPU of TX2, NVIDIA Pascal™ architecture, equipped with 256 NVIDIA CUDA Cores; CUDA version 10.0; operating system, Ubuntu 18.04; Pytorch version 1.4.0.

In the experiment, the initial learning rate was set to 1 × 10^−3^. The learning rate was attenuated once every 20 k iterations, and the attenuation coefficient was 5 × 10^−4^. The batch size was 32, and the total number of iterations was 60 k.

### 4.5. Experimental Results and Analysis

In order to verify the effectiveness of the proposed algorithm, we selected faster R-CNN, R-FCN, SSDLite [30], and different backbones of SSD models for comparison. The backbones included VGG16, ResNet101 [31], and MobileNet_V2 [30]. The specific comparison models are shown in Table 2.

#### 4.5.1. Qualitative Evaluation

**Figure 9:**
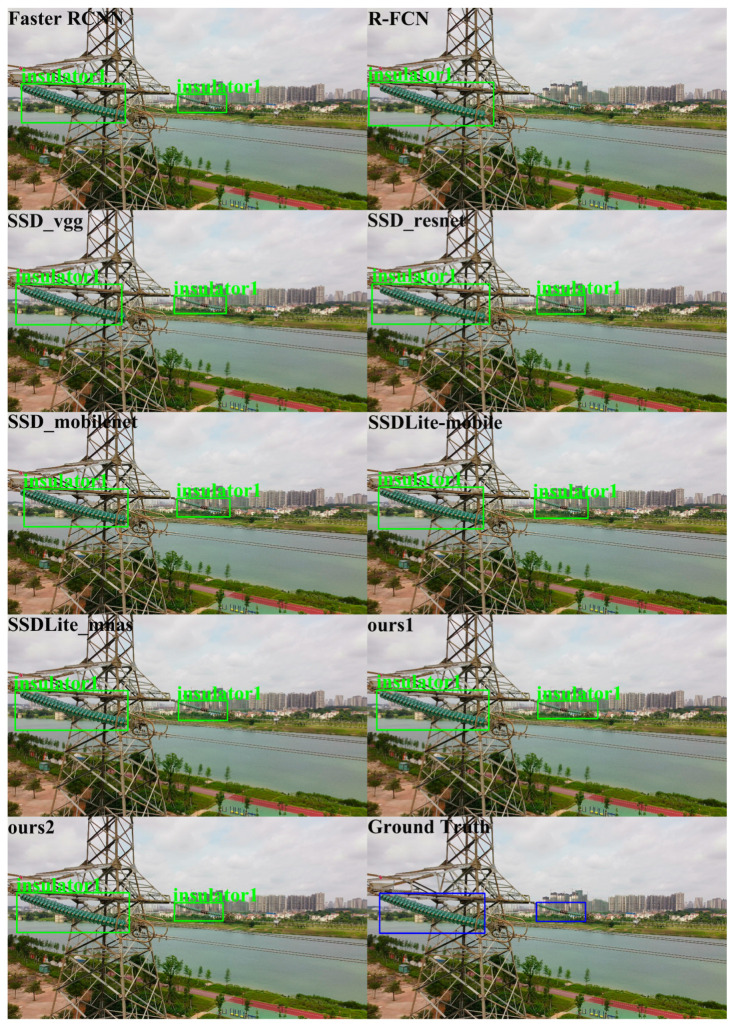
Insulator1 detection results. The blue box is the ground truth of the position of insulator1 in the image.

**Figure 10:**
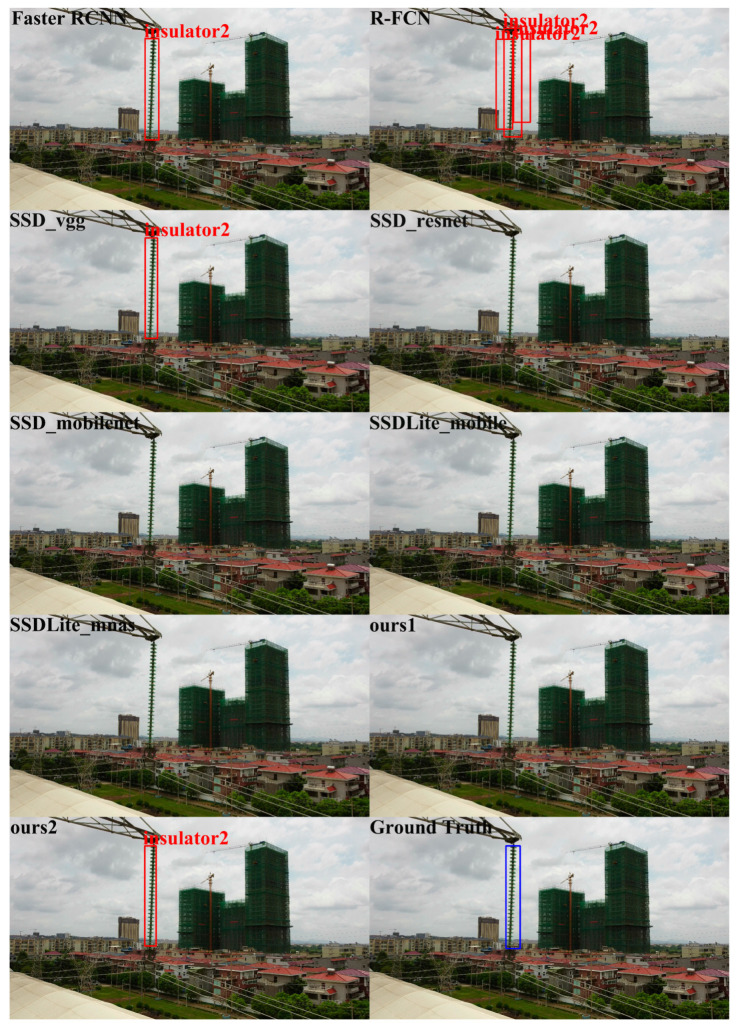
Insulator2 detection results. The blue box is the ground truth of the position of insulator2 in the image.

**Figure 11:**
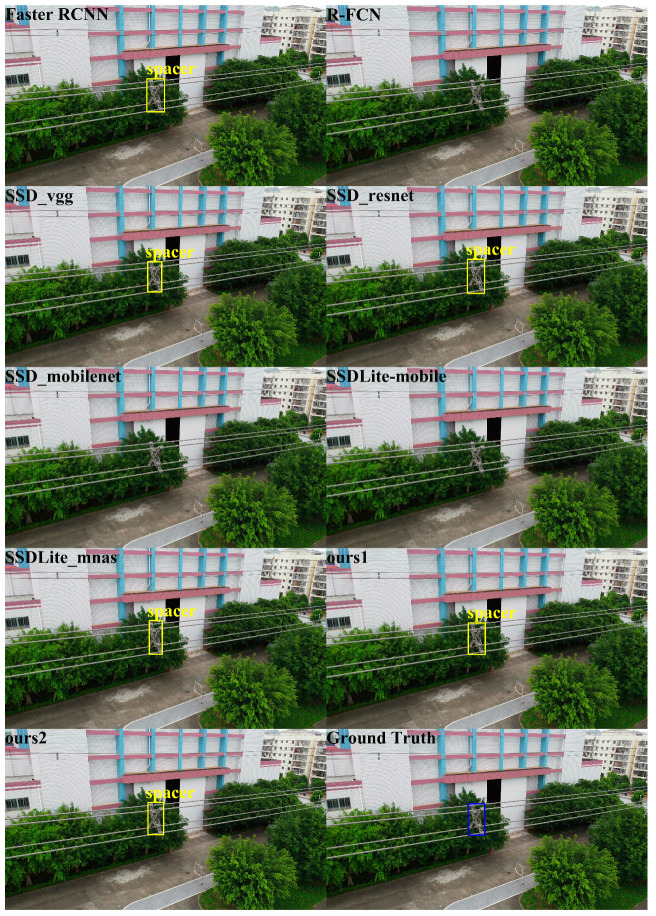
Spacer detection results. The blue box is the ground truth of the position of spacer in the image.

In order to qualitatively analyze the performance of different detectors, we show the object detection results of the different algorithms using the ISD-dataset in Figure 9, Figure 10 and Figure 11. Figure 9, Figure 10 and Figure 11 show the visualization results of different algorithms for object detection in aerial images. The detected objects are marked with boxes. The images without box markings indicate that the algorithm did not detect the object. The last subfigures of Figure 9, Figure 10 and Figure 11 represent the ground truth.

In Figure 9, for Insulator1 object detection, the nine algorithms performed well. All methods could detect the Insulator1 object, and no missed detection occurred. However, R-FCN showed overlapping detection boxes after NMS. In Figure 10, for Insulator2 object detection, SSD_vgg, Ours2 and Faster R-CNN could successfully detect the Insulator2 object. R-FCN still showed overlapping detection boxes and the other algorithms failed to detect the Insulator2 object. In Figure 11, for Spacer object detection, SSD_mobilenet, SSDLite_mobile, and R-FCN did not detect the Spacer object. The other algorithms all detected the Spacer object. From the comparison results in Figure 9, Figure 10 and Figure 11, we can see that the proposed algorithm in this paper could effectively detect Insulator1, Insulator2, and Spacer, and the detection performance was closer to ground truth. Therefore, the proposed algorithm is better than the comparison algorithms.

#### 4.5.2. Quantitative Evaluation

**Figure 12:**
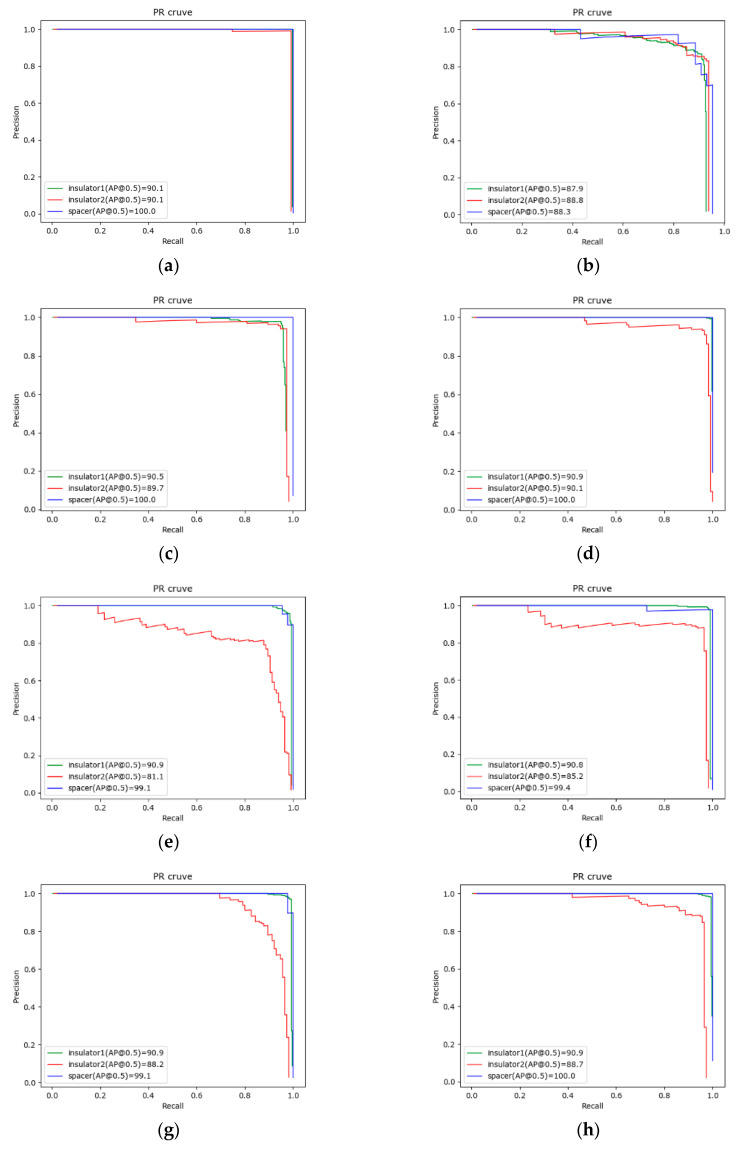

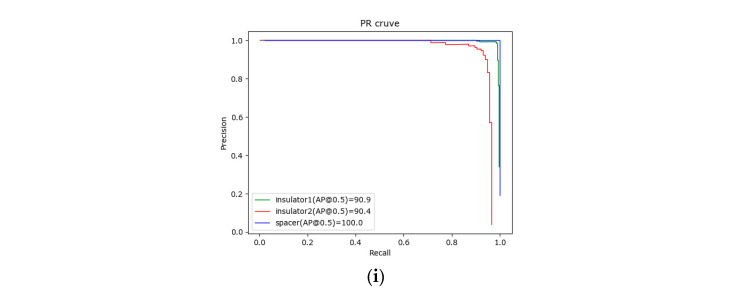
Performance comparison of different detectors: (**a**) faster R-CNN; (**b**) R-FCN; (**c**) SSD_vgg; (**d**) SSD_resnet; (**e**) SSD_mobilenet; (**f**) SSDLite_mobile; (**g**) SSDLite_mnas; (**h**) Ours1; (**i**) Ours2.

In order to quantitatively analyze the performance of compared algorithms, we use the mAP (mean Average Precision) [28] metric to evaluate the performance of the nine algorithms, and we analyzed the detection effect of different types of objects through the AP (Average Precision) metric. In addition, we counted the weight size of the models. In the detection speed experiment, we detected 300 images on the server and TX2, and we counted the detection time and total time for each image. We used the average time as the detection time for a single image, and we observed the stability of the algorithm by counting the variance of the detection time. Table 3 shows the detection accuracy of different algorithms. Table 4 shows the statistics of the detection speed of different algorithms, as well as the average detection time of a single image on the server and TX2 for each algorithm. In order to more directly compare the detection performance of different algorithms, we also drew the PR (Precision Recall) curves of each algorithm with an IoU (intersection over union) of 0.5. The specific detection results are shown in Figure 12.

The calculation methods of precision and recall are shown in Equation (15), where TP (true positive) means that the value of actual class is positive and the value of predicted class is also positive, FN (false negative) means that the value of actual class is positive and predicted class is negative, and FP (false positive) means that the value of actual class is negative and predicted class is positive. The PR curve reflects the relationship between precision and recall under different category confidence thresholds. In the PR figure, a larger area enclosed by the curve and the coordinate axis denotes better performance of the algorithm.

(15) $$Precision=\frac{TP}{TP+FP}\;Recall=\frac{TP}{TP+FN}.$$

From the comparison analysis of Table 3 and Table 4 and Figure 12, the observations below can be made.

The accuracy of faster R-CNN is 93.4% and that of R-FCN is 88.1%, whereas that of Ours2 is 0.4% higher than that of faster R-CNN and 5.1% higher than that of R-FCN. The detection time test on TX2 showed that faster R-CNN had the longest detection time of 1036 ms, and R-FCN had a detection time of 576 ms. Faster R-CNN and R-FCN cannot meet real-time requirements, and they are not suitable for deployment on a UAV.

As shown in Figure 12, our proposed algorithm’s surrounding area of the PR curve was similar to that of SSD_vgg and SSD_resnet. The mAP values of SSD_vgg, SSD_resnet, Ours1, and Ours2 were very close, whereby that of Ours2 was 0.4% higher than that of SSD_vgg and 0.1% higher than that of SSD_resnet. However, the detection time on TX2 of Ours2 was 57 ms less than that of SSD_vgg and 46 ms less than that of SSD_resnet. In terms of model size, Ours2 was 167.44 MB smaller than SSD_resnet and 47.89 MB smaller than SSD_vgg. In terms of detection time and model size, Ours2 was better than SSD_vgg and SSD_resnet. In particular, the detection time of Ours2 was the shortest on both server (15 ms) and TX2 (154 ms).

In the lightweight model, the accuracy of Ours2 was 3.5% higher than that of SSD_mobilenet, 2.0% higher than that of SSDLite_mobile, and 1.1% higher than that of SSDLite_mnas. The model sizes of SSD_mobilenet, SSDLite_mobile, and SSDLite_mnas were smaller than that of ours2, but their detection time was longer than that of ours2. Although SSDLite_mobile and SSDLite_mnas use depthwise separable convolution to reduce the number of parameters, this did not increase their detection speed. The detection time of Ours2 on TX2 was 173 ms slower than SSD_mobilenet, 166 ms slower than SSDLite_mobile, and 84 ms slower than SSDLite_mnas. Ours2 could effectively improve the accuracy and detection speed with less of an increase in model size.

The biggest difference between Ours1 and Ours2 was the feature fusion method, and the method of channel concatenate could reduce the amount of calculation. The detection time on TX2 of Ours2 was 35 ms slower than Ours1, which proves that the fusion method of Channel concatenate is better than the method of Channel plus. We can see that the variance of detection time on TX2 of Ours2 was smaller compared to other algorithms, except for faster R-CNN, showing that the detection speed of Ours2 was relatively stable.

Considering the accuracy, speed, and model size, Ours2 could achieve the best performance. Ours2 is suitable for deployment on UAVs for power inspection.

In order to test the real-time performance of Ours1 and Ours2, we employed them for video detection. The resolution of the video was 1920 × 1080. The calculation method of fps is shown in Equation (16), where Framenum is the total number of frames and elapsedTime is the elapsed time. A higher fps denotes a smoother video. We counted the fps of Ours1 and Ours2 on the server and TX2 as shown in Table 5. In video detection, Ours2 could achieve 8.27 FPS on TX2.

(16) $$FPS=\frac{framenum}{elapsedTime}.$$

**Figure 13:**
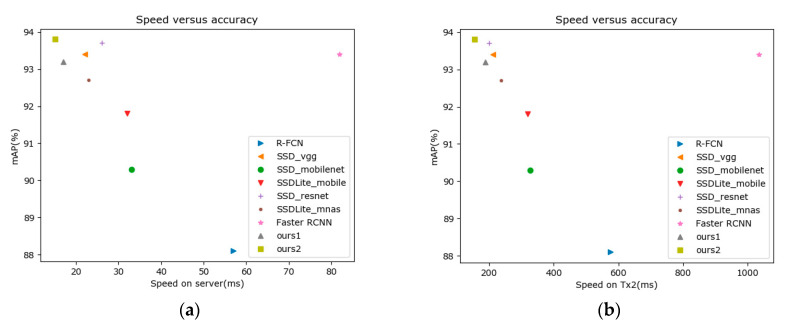
Speed versus accuracy: (**a**) test on the server; (**b**) test on TX2.

Figure 13 is a diagram of the detection accuracy and speed of our algorithm and the compared algorithms on the server and TX2. It can be seen that the two proposed algorithms are in a leading position in detection accuracy and speed, showing their superior performance.

## 5. Conclusions

This paper presented an insulator and spacer detection method based on improved SSD for UAV transmission lines. Firstly, feature extraction was performed through the MnasNet network, which can construct a lightweight network with smaller parameters and better performance for object detection. Then, a multiscale feature fusion method was used to fuse low-level features and high-level features to improve detection accuracy. The proposed object detection network was a lighter model. Finally, images of various scenes collected by a UAV were used to construct the insulator and spacer detection dataset, and experiments were conducted on the dataset to realize the identification and positioning of insulators and spacers. The experimental results verified the practicability and robustness of the improved SSD model in inspection image detection. Due to the small number of model training parameters and low model weight size, it is more suitable for deployment on embedded devices with limited computing resources. When the UAV is equipped with an embedded device, it can realize online identification without sending it back to the data terminal for detection, which can improve the inspection efficiency. Our UAV system has good scalability and can be used to detect more types of electrical components in the future.

In the future, we will collect more data to build a power inspection dataset containing more power components such as antivibration hammers, nests, connectors, and strain clamps. Furthermore, the current environment configured on the embedded device is the same as a high-performance computer environment. In the future, TensorRT will be used to optimize and accelerate the model, which can improve the detection speed and realize real-time detection.

## Figures and Tables

**Table 1:** Number of objects in each category.

Label	Insulator1	Insulator2	Spacer
Object numbers	1485	613	199

**Table 2:** Models used in the experiment. Backbone: The basic network used for feature extraction. Main Modules: The basic framework, backbone and feature fusion method of the algorithm.

Simplified Name	Backbone	Main Modules
Faster R-CNN	ResNet-101	Faster R-CNN(ResNet-101) ^1^
R-FCN	ResNet-101	R-FCN(ResNet-101)
SSD_vgg	VGG-16	SSD(VGG-16)
SSD_resnet	ResNet-101	SSD(ResNet-101)
SSD_mobilenet	MobileNet_V2	SSD(MobileNet_V2)
SSDLite_mobile	MobileNet_V2	SSDLite (MobileNet_V2)
SSDLite_mnas	MnasNet	SSDLite (MnasNet)
Ours1	MnasNet	SSD (MnasNet + Feature fusion + Channel plus) ^2^
Ours2	MnasNet	SSD (MnasNet + Feature fusion + Channel concatenate) ^3^

^1^ The basic framework of the algorithm is Faster R-CNN. The backbone is ResNet-101 and there is no feature fusion module. ^2^ The basic framework of the algorithm is SSD. The backbone is MnasNet and the feature fusion method of channel plus is used. ^3^ The basic framework of the algorithm is SSD. The backbone is MnasNet and the feature fusion method of channel concatenate is used.

**Table 3:** Detection accuracy of different algorithms. Note that the best results are bold-faced.

Method	Insulator1 AP (%)	Insulator2 AP (%)	Spacer AP (%)	mAP (%)
Faster R-CNN	90.1	90.1	**100.0**	93.4
R-FCN	87.9	88.8	88.3	88.1
SSD_vgg	90.5	89.7	**100.0**	93.4
SSD_resnet	**90.9**	90.1	**100.0**	93.7
SSD_mobilenet	**90.9**	81.1	99.1	90.3
SSDLite_mobile	90.8	85.2	99.4	91.8
SSDLite_mnas	**90.9**	88.2	99.1	92.7
Ours1	**90.9**	88.7	**100.0**	93.2
Ours2	**90.9**	**90.4**	**100.0**	**93.8**

**Table 4:** Statistics of detection time of different algorithms; server_time: the average detection time of a single image on the server; TX2_time: the average detection time of a single image on TX2; TX2_vartime: variance of the detection time of the image on TX2. Note that the best results are bold-faced.

Method	Model Size (MB)	Server_Time (ms)	TX2_Time (ms)	TX2_Vartime (ms)
Faster R-CNN	360.07	82	1036	**40**
R-FCN	383.42	57	576	58
SSD_vgg	91.62	22	211	66
SSD_resnet	211.17	26	200	70
SSD_mobilenet	40.38	33	327	76
SSDLite_mobile	**11.91**	32	320	76
SSDLite_mnas	19.77	23	238	70
Ours1	43.58	17	189	54
Ours2	43.73	**15**	**154**	45

**Table 5:** The fps of Ours1 and Ours2.

Model	Server (fps)	TX2 (fps)
Ours1	10.64	2.79
Ours2	36.85	8.27
